# Do syllables play a role in German speech perception? Behavioral and electrophysiological data from primed lexical decision

**DOI:** 10.3389/fpsyg.2014.01544

**Published:** 2015-01-12

**Authors:** Heidrun Bien, Jens Bölte, Pienie Zwitserlood

**Affiliations:** ^1^Centre for Psychiatry, Wolfson Institute of Preventive Medicine, Queen Mary University of LondonLondon, UK; ^2^Institute for Psychology, Westfälische Wilhelms-University MünsterMünster, Germany

**Keywords:** speech perception, form priming, ERPs, lexical access, lexical decision, syllables, fragment priming, German language

## Abstract

We investigated the role of the syllable during speech processing in German, in an auditory-auditory fragment priming study with lexical decision and simultaneous EEG registration. Spoken fragment primes either shared segments (related) with the spoken targets or not (unrelated), and this segmental overlap either corresponded to the first syllable of the target (e.g., /teis/ – /teisti/), or not (e.g., /teis/ – /teistləs/). Similar prime conditions applied for word and pseudoword targets. Lexical decision latencies revealed facilitation due to related fragments that corresponded to the first syllable of the target (/teis/ – /teisti/). Despite segmental overlap, there were no positive effects for related fragments that mismatched the first syllable. No facilitation was observed for pseudowords. The EEG analyses showed a consistent effect of relatedness, independent of syllabic match, from 200 to 500 ms, including the P350 and N400 windows. Moreover, this held for words and pseudowords that differed however in the N400 window. The only specific effect of syllabic match for related prime—target pairs was observed in the time window from 200 to 300 ms. We discuss the nature and potential origin of these effects, and their relevance for speech processing and lexical access.

## Introduction

In a familiar language, listeners perceive speech as a sequence of discrete and meaningful units, though the spoken input consists of a continuous and often noisy signal. Speakers provide few reliable cues on how to organize this continuous signal into units of meaning. Speech is highly variable between, and even within, speakers. Moreover, speech segments (such as phonemes) partially overlap due to coarticulation, and can vary widely depending on the phonemic context. A question that is still not fully resolved is how this variable and noisy input is mapped onto word forms and meaning. One idea is that the input is mapped onto stored sublexical units, which aid access to lexical representations of word form. Among the candidates proposed as mediators between the acoustic input and the lexicon, two have received special attention: phonemes and syllables (Cutler et al., 1986; Dumay et al., 2002; Zwitserlood, 2004).

There is quite some evidence for phoneme-sized prelexical representations (cf. Hickok and Poeppel, 2007; Obleser and Eisner, 2009), including our own work with MEG and EEG (Bien et al., 2009; Bien and Zwitserlood, 2013). Obviously, sublexical phonemic units aid in abstracting away from the noisy input. According to word-recognition models in which the speech input, in terms of features and/or phonemic segments, is continuously mapped onto lexical word-form representations, irrespective of where words begin or end, such units suffice for lexical access and selection (cf. McClelland and Elman, 1986; see Christiansen and Chater, 2001). Other models of spoken-word recognition assume that potential word onsets are important for lexical access (cf. Marslen-Wilson, 1987; Norris and McQueen, 2008). Detection of potential word-onsets presupposes segmentation or marking of incoming speech. Various cues have been proposed to signal potential onsets, such as transitional probabilities between consecutive segments (Saffran et al., 1996), phrase-boundary cues (Christophe et al., 2004), syllable duration and stress (Tyler and Cutler, 2009; Langus et al., 2012) or a combination of such cues (Mattys et al., 2005). Syllabic boundaries may also provide valuable cues for lexical access (Content et al., 2001; Cutler et al., 2001; Zwitserlood, 2003, 2004). Interestingly, word-initial syllables showed a more pronounced N100 in the EEG, compared to word-medial or final syllables (Sanders and Neville, 2003), and a study with newly learned pseudowords showed a similar N100 effect (Sanders et al., 2002).

Early evidence for the role of syllables in speech perception has been obtained in studies using monitoring paradigms (e.g., Mehler et al., 1981). In these studies, a (visually or auditorily presented) fragment precedes a spoken word, and participants have to press a button whenever the fragment is contained in the word (e.g., PA/PAL in the French word “palace”). In this paradigm, known as fragment or sequence monitoring (cf. Frauenfelder and Kearns, 1996), reactions are often faster when the fragment corresponds to the first syllable of the spoken word (e.g., PA – /palace/) than when not (e.g., PAL in /palace/). This has been taken as evidence that listeners syllabify the incoming speech signal, and for syllable-based mental representations that mediate access to the lexicon (cf. Bradley et al., 1993). Using fragment-monitoring paradigms, syllable effects have been demonstrated in various languages, such as French, Spanish, Italian, Dutch, and Portuguese (Mehler et al., 1981; Morais et al., 1989; Bradley et al., 1993; Zwitserlood et al., 1993; Dumay and Content, 2012; see Floccia et al., 2012, for an excellent overview). The evidence is mixed for English (Cutler et al., 1986; Bradley et al., 1993).

Syllables do not seem to be good candidates for prelexical processing across all languages, and there is good reason to expect varying results in different languages. Languages differ greatly in the extent to which syllable boundaries are clear and unambiguous, as well as in the regularity of syllable structure (Bradley et al., 1993). English includes many ambisyllabic segments (i.e., segments that are part of two adjacent syllables). According to Bradley et al. (1993), a preponderance of ambisyllabicity may render syllabic segmentation inadequate. Consequently, other segmentation aids have been proposed (e.g., Cutler et al., 1986) for English listeners. Cutler et al. (1997) argue that listeners exploit the rhythmic structure characterizing their language in order to segment the speech signal. Cutler et al. (1997) assume that the viability of the syllable as an aid in speech segmentation corresponds to the basic prosodic structure of natural languages. In stress-timed languages such as English, listeners use stress as a segmentation cue, whereas in syllable-timed languages such as French and Spanish, listeners use syllabic boundary information. German and Dutch are stress-timed, but in Dutch, a language with high ambisyllabicity, syllabic effects have been observed (Zwitserlood et al., 1993). It is noteworthy that the interpretation that syllabic effects provide evidence for syllable-sized prelexical units of speech has been largely abandoned in the last decade or so. The more general view that syllable-boundary information—among various other cues—aids the parsing of speech input for lexical access has been proposed instead (Content et al., 2001; Cutler et al., 2001; Dumay et al., 2002; Zwitserlood, 2003, 2004).

Fragment or sequence monitoring is not the only paradigm with which effects of syllables in speech comprehension can be examined. An alternative is fragment priming, used to investigate lexical activation and access in spoken-word processing. Fragment priming can involve form relatedness (/kaep/ – captain) or semantic relatedness (/kaep/ – ship) between primes and targets (see Zwitserlood, 1989, 1996). The paradigm can be cross-modal, with spoken fragments and visually presented target words (similar to most fragment-monitoring studies) or unimodal, with spoken fragments and spoken targets. Form-related spoken fragments that match the target (e.g., /stri:/ – street) facilitate target processing relative to mismatching fragments (e.g., /stra:/ – street; cf. Marslen-Wilson, 1993). The paradigm has not been used often to investigate particular aspects of the fit between fragments and targets. Exceptions are the studies reported by Friedrich and colleagues. Friedrich et al. (2004a), for example, showed clear EEG correlates of segmental overlap between fragments and target words (e.g., /dra/ – dragon vs. /hun/ – dragon). They also investigated particular aspects of form overlap, for example shared place of articulation of (differing) initial consonants of fragments and targets (Friedrich et al., 2008; Schild et al., 2012), or pitch (Friedrich et al., 2004b).

In the present study, we used fragment priming with lexical decision to study effects of syllabic match in German. Note that German has not been studied before with respect to a specific role for syllables in speech perception. There are studies in German on the role of syllables in visual word processing that demonstrate negative effects when the first syllable of a target word is of high frequency. This inhibition is evident in lexical decision (Conrad and Jacobs, 2004) and in early (200–300 ms) event-related components of EEG (Hutzler et al., 2004). The interpretation is that words are parsed into phonologically defined syllables during reading (cf. Conrad et al., 2007). However, interesting these results are, they provide no direct evidence for a similar role for syllables during speech processing. The fragment-priming studies with EEG by Friedrich and colleagues, all conducted in German, showed positive effects of segmental overlap. From the examples, the fragment primes seem to correspond to the first syllable of the target (e.g., /trep/ – treppe or /kan/ – kante), but it remains unclear whether there is a specific advantage of syllable-sized primes (Friedrich, 2005; Friedrich et al., 2009; Schild et al., 2012). Note also, that most studies employed a crossmodal paradigm (but see Friedrich et al., 2009; Schild et al., 2012), which seems less suitable to pick up early, or prelexical, effects of overlap between fragments and targets. We decided to use the unimodal priming variant (auditory fragments, auditory targets), because effects of syllabic match may be prelexical and/or modality-specific. We collected behavioral data—lexical decision latencies to spoken words and pseudowords primed by spoken fragments—and simultaneously recorded event-related potentials (ERPs).

Our participants performed lexical decisions on auditory stimuli (e.g., “lustig” – funny), preceded by related (e.g., /lus/) or unrelated (e.g., /tra/) auditory primes. Note that we used prime fragments spoken in isolation, not excised from longer stimuli. This was done to avoid particular information that is present in fragments that are cut out of words. One type of information comes from coarticulation of adjacent segments, another from subtle cues that signal syllabic boundaries. For example, Zwitserlood (2004) demonstrated for Dutch that fragments such as /mark/ cut out of /marker/ contain information about the syllabic boundary between /mar/ and /ker/. In fact, such cues drove the syllable-match effects obtained in that study. As our main aim was to study the role of syllables in speech processing at a level that abstracts away from particular cues provided in running speech, we opted for fragments spoken in isolation. Evidently, no solution is ideal, since fragments spoken in isolation tend to be longer than corresponding parts of longer words (Salverda et al., 2003). But note also that positive effects of overlap have been found before, with spoken prime-target stimuli that did not overlap completely (e.g., the French pseudoword “lurage” priming the target word “tirage”; Dumay et al., 2001).

Our predictions for the reaction time latencies were as follows. If related fragments activate corresponding words in the mental lexicon, lexical decision should be facilitated, compared to unrelated fragments. Crucially, if syllables play a role in German speech perception, related primes that precisely match the initial syllable, as in /lus/ – /lus.tig/ (funny), and /lust/ – /lust.los/ (listless; the dot marks the syllable boundary) should be superior to primes that match an equivalent number of initial phonemes but do not match the first syllable (e.g., /lus/ – /lust.los/, and /lust/ – /lus.tig/).

We also manipulated the relatedness between fragments and targets in the pseudoword trials. Note that this is hardly ever done, because pseudoword trials, necessary for the lexical decision task, are often considered uninteresting for other purposes (but see Friedrich et al., 2004a). Our pseudoword trials differed in critical aspects from the word trials. First, the fragments used with pseudoword targets did not correspond to existing German words or morphemes (e.g., wos, zas, limp, wost). The idea was to assess, with the pseudoword sets, effects of segmental overlap—even of syllable-sized segmental overlap—under conditions that minimize lexical contributions to the effects. For the same reason, the pseudoword targets were not very similar to existing words (e.g., wosteck, limpal, zastig) and many of the fragment primes did not even correspond to existing syllables (in particular the long primes). We explicitly avoided pseudowords consisting of two existing morphemes, such as “lustbar” or “mutung” that could exist but happen not to occur in the language (see Bölte et al., 2009, for EEG data on such stimuli). Thus, the pseudoword stimuli, in addition to their purpose for lexical decision, were used to assess the contribution of (syllable-sized) form overlap to the processing of spoken stimuli with as little lexical contribution as possible. Comparing word and pseudoword targets with respect to effects of (syllabic) match is informative with respect to the locus of these effects (lexical, prelexical; existing vs. possible syllables).

A comparison of behavioral and ERP data may shed light on the automaticity of potential effects and on their dependence on lexical processing, because EEG data are informative about the time course of effects. Based on the literature, we expected effects of relatedness in the EEG data. Both studies with auditory-auditory priming (Friedrich et al., 2009; Schild et al., 2012) revealed modulations of early (before 200 ms) components (N100, T-complex) by relatedness, as well as effects in the P350, a component sensitive to the goodness-of-fit between fragments and targets, reflecting lexical activation (Pylkkänen and Marantz, 2003; Friedrich et al., 2009). Effects in the N400 range are taken to reflect lexical processing, that is, the fit between the input provided by the prime fragment and the lexical representation of the spoken target. As for syllabic match, there are no data to guide our predictions. Early modulations of the EEG should be evident when syllabic match plays a role during early phases of speech processing and lexical access. Modulations in the N400 domain would rather point to late, lexical effects, so a difference between word and pseudowords is expected here.

## Methods

### Participants

Seventeen students of the Westfälische-Wilhelms Universität Münster, Münster, Germany (four males) with mean age 21 years (*SD* = 3.4, aged 19–31 years) took part in the experiment. All participants were native speakers of German and right-handed according to the Edinburgh Handedness Inventory (Oldfield, 1971). None reported any (history of) hearing loss or neurological problems. They received 12€ or course credit for their participation.

### Materials

All stimuli were spoken by a trained female native speaker of German and recorded using a high-quality microphone and a digital recorder (M-AUDIO microtrack 24/96) with a sampling rate of 44.1 Hz. For stimulus extraction and editing, we used the software packages *Praat* (Boersma and Weenink, 2010; version 5.0.23) and *CoolEdit* (CoolEdit 2000 v1.1).

The experiment contained 37 pairs of word targets, all of which were bisyllabic with clear syllable boundaries (e.g., lus.tig^1^, funny, and lust.los, dull). All word targets were morphologically complex, derived words. The targets of each pair differed in length (Long vs. Short; for durations and other relevant matching parameters see Appendix 1 in Supplementary Material), shared the initial morpheme (“lust,” delight), but differed in the length of their first syllables, due to re-syllabification of these morphemes (e.g., lust.los vs. lus.tig). As shown in Table 1, Short and Long targets were combined with two form-related (e.g., /lus/, /lust/) and two unrelated (e.g., /tra/, /trag/) spoken primes. Prime fragments were not excised from target words but recorded separately as monosyllabic stimuli. The two targets of a pair (e.g., lust.los – lus.tig) were combined with the same four primes, forming a set of eight trials with which stimulus properties and priming effects can be disentangled. In half of the trials, prime and target were related (e.g., /lus/ – /lust.los/), in half, they were unrelated (e.g., /trag/ – /lust.los/). Given that primes in the two related conditions differed in length, related, and unrelated primes were matched for length (/lus/ – /tra/; /lust/ – /trag/; related primes were some 20 ms shorter than unrelated ones), as well as with for pitch and intensity (see Appendix 1 in Supplementary Material). Unrelated primes did not occur as related primes elsewhere.

**Table 1:** **Example of materials**.

**Lexicality**	**Syllabic match**	**Unrelated**	**Related**	**Target**	**Target**
Word	+	/trag/	/lust/	Long	lust.los
	−	/tra/	/lus/		lust.los
	+	/tra/	/lus/	Short	lus.tig
	−	/trag/	/lust/		lus.tig
Pseudoword	+	/womp/	/bent/	Long	^*^bent.lok
	−	/wom/	/ben/		^*^bent.lok
	+	/wom/	/ben/	Short	^*^ben.to
	−	/womp/	/bent/		^*^ben.to

Note: A dot marks a syllable boundary. An asterisk marks a pseudoword.

Crossed with the factor Relatedness was the factor Syllabic Match, which is a dummy variable for unrelated trials (see below). In half of all trials, indicated by a “+” sign in Table 1, the structure and segments of the prime matched the target-initial syllable (e.g., /lus/ – /lus.tig/, funny). In the other half, indicated by a “−” sign, despite shared phonemes, there was no syllable-structure match (e.g., /lus/ – /lust.los/, listless). In related trials, the target always contained the prime. Only in half of the related trials did the prime exactly match the initial syllable of the target (e.g., /lus/ – /lus.tig/; /lust/ – /lust.los/). In the remaining half, the related prime was either shorter (e.g., /lus/ – /lust.los/) or longer (e.g., /lust/ – /lus.tig/) than the initial syllable. As is standard in control trials, the unrelated primes were in all aspects unrelated to their targets. With very few exceptions, this also concerned the syllabic skeleton of control primes and targets, so that the syllabic structure (CV, CVC, CCV, CCVC, and so on) of unrelated and related primes differed. Appendix 4 in Supplementary Material contains all word and pseudoword stimuli.

While Target Length and Syllabic Match are crossed for related pairs, there remains an imbalance with respect to the morphological status of the prime fragments. The longer prime fragments always corresponded to the stem morpheme of both target words. All stimuli had a transparent semantic relation between the stem morpheme and both targets. Given that many of the short targets were verbs, the long primes constituted the verbal stem of these stimuli (e.g., /greif/ in /greifen/, to grasp) and were potentially even more closely related to these verbs than to the derived longer words (e.g., /greifbar/ – graspable). Note also that in some cases, the short, CVC prime fragments corresponded to an existing morpheme (e.g., /lau/, tepid, from the target pair /lauf.band/ – /lau.fen/, treadmill, walk), but these morphemes were always semantically unrelated to the targets.

Another 37 sets of pseudowords (non-existing but phonotactically legal strings) were added for the lexical decision task. These sets occurred in similar conditions as the word sets. The pseudoword targets were created to be dissimilar to existing words while conforming to the phonotactic constraints of German. Almost without exception (e.g., /ris/ – Riss, crack), the fragment primes of pseudoword targets did not correspond to existing words or root morphemes of German. Note that syllabic overlap in fragment—pseudoword pairs is defined via the syllabic structure of the pseudoword targets and often involves non-existing, but possible syllables of German.

With 74 experimental sets (37 word sets, 37 pseudoword sets), and eight prime-target pairings per set, the number of experimental trials presented to each participant was 592. These trials were distributed over four blocks, such that there was no repetition of a prime or a target within a block. Using Latin square designs, conditions were evenly distributed over blocks, and block order was balanced between participants. To reduce the proportion of trials with prime-target overlap, we added 144 filler trials with 72 filler targets (36 words, 36 pseudowords) each combined with two different, unrelated prime fragments. None of these filler primes or targets was used in an experimental trial. The experiment started with seven additional warm-up trials of similar structure.

### Procedure

Participants were individually tested, comfortably seated in front of a computer screen (Samsung SyncMaster 2233RZ, 22″, 120 Hz refresh rate, 1680 × 1050 pixel, 32 bit color depth) and a button box (Response Pad, Model RB – 830, producer Cedrus Corporation). They were instructed before and kept informed during the testing phase. Upon informed consent, the EEG-cap was positioned on the participant's head and two researchers simultaneously prepared the 64 electrodes. The experiment was controlled using the software *Presentation* (producer Neurobehavioral Systems, version 14.1.). To minimize artifacts, we asked participants to keep looking at a fixation cross at the center of the screen, and to blink and move as little as possible during trials. The auditory stimuli were presented via Sennheiser IE6 in-ear headphones and participants were allowed to adjust the volume to their individual preferences.

**Figure 1:**
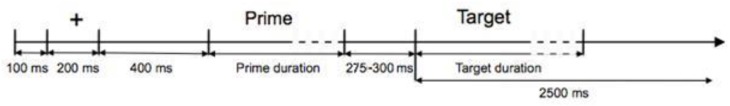
**Time course of a trial**.

At the beginning of each trial (see Figure 1), a black fixation cross (Courier New, 48 pt) was presented at the center of the white screen where it remained until the end of the trial. Four hundred milliseconds after the appearance of the fixation cross, the auditory prime was presented, followed with a temporal jitter of 275–300 ms by the auditory target. Participants could provide their lexical decision from target onset onwards, pressing one of two buttons using their index fingers. For individual participants, left-right button assignment to word and pseudoword decisions remained the same throughout the experiment, between participants it was balanced. Participants were instructed to decide as quickly and accurately as possible. Decisions and the reaction times were recorded starting from target onset.

We followed standard EEG recording and analysis procedures (Picton et al., 2000). The EEG was recorded continuously from 64 Ag/AgCl electrodes using a WaveGuard cap (ANT Software B.V., The Netherlands) connected to a high input impedance amplifier (ANT ASA-lab amplifier, digital low-pass FIR-filter, cut-off frequency = 0.27 ^*^sampling rate). Two additional electrodes were placed on the outer left and right canthi and another two above and below the left eye to monitor eye movements. Impedances were kept below 10 kΩ. A high-impedance amplifier in combination with actively shielded electrode caps enables clear signals even with high electrode impedances (Ferree et al., 2001). The EEG was recorded with a sampling rate of 256 Hz using an average reference (Dien, 1998). Triggers were set to the beginning of the target stimulus.

Every 1.5–2 min there was a short break of 10–15 s. Every 7.5–9 min there was a longer break of 1.5–2 min. During breaks, participants were allowed to move freely. The breaks were counted down by seconds on the screen, enabling participants to resume a comfortable position before the start of the consecutive trials. With 743 trials and the various breaks, the experiment lasted approximately 70 min. Including instructions, application and removal of the EEG-cap, a session took about 2 h.

## Results

### Behavioral results

Trials with reaction times (RTs) above 2500 ms (0.2%) were excluded as were trials with incorrect lexical decision (1.6%). Error rates were below 5% for all participants. Three of the 74 sets were excluded from analyses due to high error rates for one of the targets. For the remaining 71 sets, we performed an outlier correction, excluding RTs outside two standard deviations from the mean per participant and condition (5.5%). The excluded trials as well as the remaining errors were evenly distributed over conditions and not analyzed further.

Overall, mean RTs were shorter for word (963 ms) than for pseudowords targets (1036 ms). This is a common finding in lexical decision and indicates that the pseudowords were not easy to reject as existing words (compared to phonotactically illegal stimuli such as “prlaspkusx”) even though they turned into pseudowords well-before word offset (see Table 2 for means and SD per condition). We ran Three-Way repeated measures ANOVAs with Relatedness (related vs. unrelated), Syllabic Match (present/absent in related conditions) and Target Length (short vs. long), separately for reaction times to word and pseudoword targets. The ANOVA using RT toward words revealed a significant effect of Target Length [*F*_(1, 16)_ = 10.200, *p* = 0.006, η^2^_*p*_ = 0.389]. Not surprisingly, short targets (mean: 953 ms, SE: 35) yielded faster RTs than long targets (mean 972 ms, SE: 35). The interaction of Relatedness and Syllabic Match proved to be significant [*F*_(1, 16)_ = 27.098, *p* = 0.001, η^2^_*p*_ = 0.629]. This interaction showed that reactions to targets were facilitated when the related prime matched their first syllable (25 ms), but not when preceded by a prime that merely shared initial segments with the target (−13 ms). The three-way interaction [*F*_(1, 16)_ = 7.969, *p* = 0.012, η^2^_*p*_ = 0.332] was also significant. All other effects were not significant (*F* = 1.481, *p* = 0.214, η^2^_*p*_ = 0.085).

**Table 2:** **Mean reaction times in ms and SD (in parentheses) as a function of conditions**.

**Lexicality**	**Relatedness**	**Syllabic match**	**Target length**	
			**Short**	**Long**
Word	Related	+	930 (132)	967 (153)
		−	971 (147)	972 (142)
	Unrelated	(+)	965 (144)	980 (146)
		(−)	947 (164)	970 (145)
Pseudoword	Related	+	1023 (155)	1069 (183)
		−	1013 (153)	1066 (175)
	Unrelated	(+)	1016 (167)	1046 (190)
		(−)	999 (176)	1054 (173)

The three-way interaction was evaluated by means of *t*-tests, contrasting mean RTs to targets after related and unrelated primes. There was a clear 35 ms priming effect of short primes followed by their matching targets [e.g., /luf/ – /luf.tig/, *t*_(16)_ = 2.606, *p* = 0.009]. The smaller effect (13 ms) for long primes and their syllable-matching targets (e.g., /luft/ – /luft.los/) failed significance [*t*_(16)_ = 1.500, *p* = 0.076]. There was no facilitation of lexical decision latencies in cases of mere phonological overlap. When the fragment primes matched the segments of the target but not its first syllable (e.g., /luft/ – /luf.tig/) the short targets revealed a numerical 24 ms interference effect which was not significant [*t*_(16)_ = 1.634, *p* = 0.061] despite the fact that the long fragment prime corresponds to the stem morpheme. The condition with long targets (e.g., /luf/ – /luft.los/) showed no effect (−2 ms, *t* < 1).

The ANOVA using RT toward pseudowords yielded a different pattern. Only the factors Relatedness [*F*_(1, 16)_ = 5.157, *p* = 0.037, η^2^_*p*_ = 0.244] and Target Length [*F*_(1, 16)_ = 44.304, *p* = 0.001, η^2^_*p*_ = 0.735] proved significant. Short targets (mean: 1013, SE: 39) attracted faster RTs than long targets (mean: 1059, SE: 43). Pseudoword targets were responded to faster when preceded by unrelated primes (mean: 1029, SE: 42) than by related ones (mean 1043 ms, SE: 40). All other effects were not significant (*F* = 2.736, *p* = 0.118, η^2^_*p*_ = 0.146).

To summarize the reaction-time data: No facilitation was evident for pseudowords preceded by related fragments; inhibition was observed instead. In contrast, there was facilitation for word targets preceded by fragments that matched their first syllable. This main effect of syllabic match was strongly evident for short fragments and short targets, and just failed significance for long fragments/long targets. This is surprising, given that the matching fragments (e.g., lust) of long targets (e.g., lust.los) correspond to the syllable as well as to the stem morpheme of the target. In Dutch, this double overlap resulted in larger effects than mere syllabic overlap evident with short prime fragments, but note that these data come from fragment monitoring, not from priming (Zwitserlood, 2003). In contrast to syllabic match between fragments and targets, the cases of syllabic mismatch—but still providing phonemic overlap—showed no priming.

The difference between word and pseudoword targets can be interpreted in two ways. First, it is possible that the existence of the primes as syllables of the language, for example as members of a mental syllabary (Levelt and Wheeldon, 1994), is a prerequisite for priming by syllable-sized fragments. If this plays a role, we would have expected a difference between the long and short pseudoword primes, since about half of the short primes were (rather infrequent) syllables of German, but the long ones were not.

An intriguing finding is the lack of priming with long fragments and short word targets, when related primes mismatch the first syllable but still constitute targets' stem morpheme. In fact, this condition with syllabic mismatch showed numerical interference instead of facilitation. In contrast, the pseudoword conditions—in the first-block ANOVA—showed interference in cases of syllabic match, that is, in conditions that produce facilitation for word targets. A possible interpretation for this reversal of effects focuses on the nature of the targets, word or pseudowords. The only positive syllabic effects occurred with word targets, interference was observed when the target was a pseudoword, in particular for related primes that syllabically matched their target. This suggests a sensitivity to the syllabic structure of the targets that results in speeded word decisions and slowed pseudoword decisions. A likely locus for such a pattern is a postlexical, strategic one.

Auditory lexical decision thus seems to tap into late effects of syllabic match and may not be ideally suited to investigate the role of syllables in pre-lexical and early lexical speech processing. For this, EEG data might be better suited.

### ERP results

EEG-data were analyzed using a combination of ASA (ANT, The Netherlands), EEGLAB (version 12.0.2.06b, Delorme and Makeig, 2004; MATLAB 2012b) and ERPLAB (version 4.0.2.3, Lopez-Calderon and Luck, 2014). EEG-data were filtered using a half-power Butterworth bandpass filter (0.1–20 Hz, 24 db/oct) based on the FFT-method. Ocular artifacts were corrected using a PCA-approach (Ille et al., 2002). Remaining artifacts were detected using a ±75 μV threshold. There were on average 11% errors in the word condition and 10% errors in the pseudoword condition (see Appendix 2 in Supplementary Material for a complete compilation). Artifact-free trials with correct responses were averaged using epochs of 700 ms length, time locked to target onset, with a 200 ms pre-stimulus baseline. We formed six regions of interest (anterior central: Fp1, Fpz, Fp2, AF3, AF4, F1, Fz, F2; anterior left: F7, F5, FT7, FC5, FC3, T7, C5, C3; anterior right: F8, F6, FT8, FC6, FC4, T8, C6, C4; posterior central: CP1, CPz, CP2, P3, Pz, P4, POz, Cz; posterior left: TP7, CP5, P7, P5, PO7, PO5, PO3, O1; posterior right: TP8, CP6, P8, P6, PO8, PO6, PO4, O2). These six ROIs constituted the variables LR-axis (left, central, right) and A-P (anterior, posterior).

The EEG-data quality for two participants was too low to be included in further analyses. Mean voltage was calculated in a number of time windows that were shown to be of interest in unimodal fragment priming (cf. Friedrich et al., 2009; Schild et al., 2012). The first two windows (80–200 ms, including the N100) and (200–300 ms) are taken to reflect early modality-specific processing of speech input (cf. Friedrich et al., 2009). Next, data were analyzed in time windows that were shown to be relevant for auditory-auditory priming with matching or mismatching word fragments: the 300–400 ms window, including potential P350 effects, and the 280–500 ms window, including the N400, where lexical effects are expected next to effects of the overlap between fragments and targets.

EEG data for correct reactions in lexical decision were included, and all ANOVAs (except the last one) had the following within-factors: Syllabic Match (present vs. absent in related conditions), Relatedness (related vs. unrelated), Target Length (short, long), A-P (anterior, posterior), and LR-axis (left, central, right). The ANOVA on the N400 time window included a different electrode selection. Word and pseudoword targets were analyzed separately. Effects including more than two levels are reported only if they remained significant after Greenhouse-Geisser correction; effects of electrodes (A-P, LR-axis) are reported only when interacting with manipulated factors.

### Time window 100–200 ms

The ANOVAs showed no significant effects of any of the variables, nor interactions between them, in this time window. This held for word and pseudoword targets alike.

### Time window 200–300 ms

**Figure 2:**
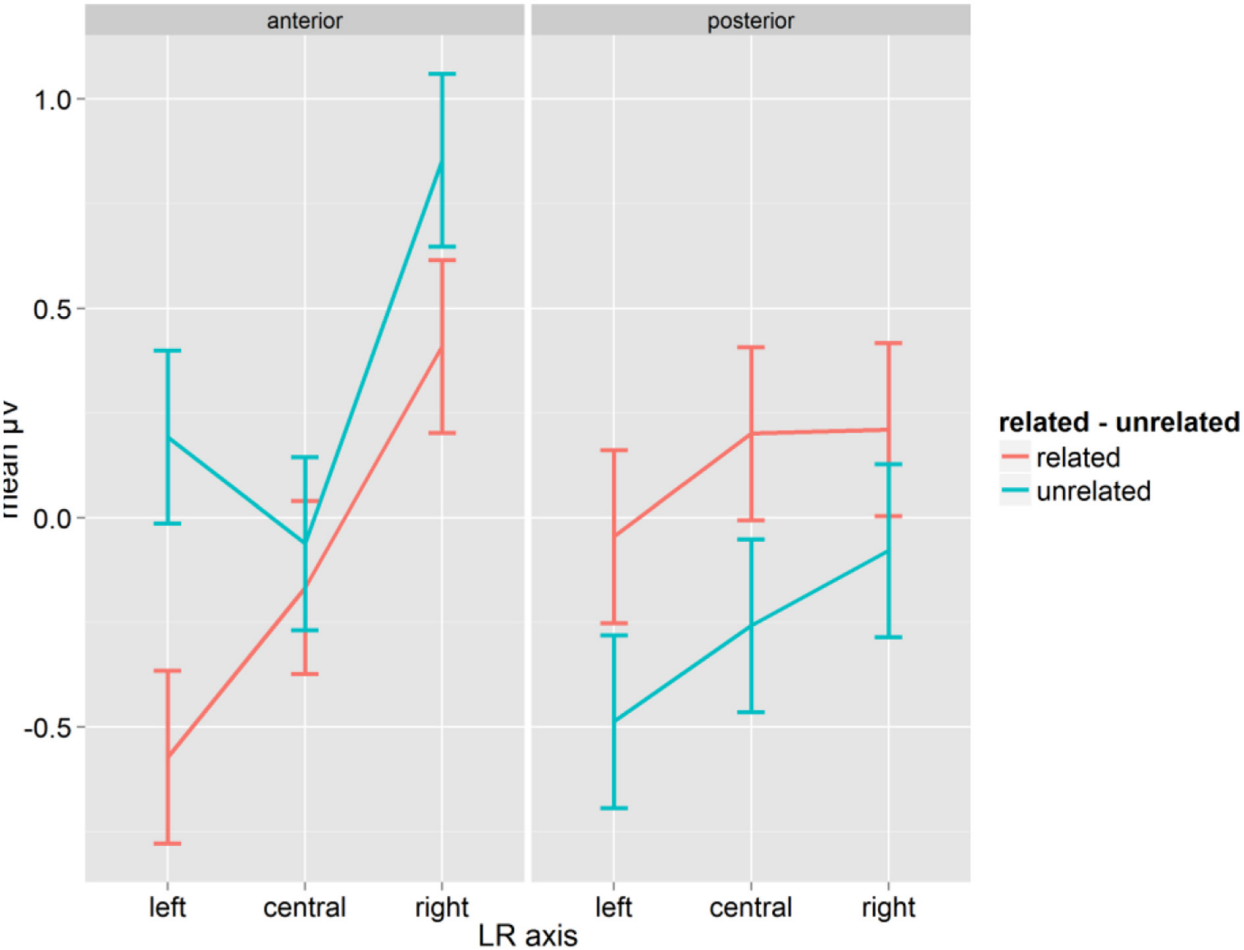
**Mean μV for words as a function of conditions for the time-window 200–300 ms; error bars represent Fisher's least significant differences**.

The repeated-measure ANOVA with Syllabic Match, Relatedness, Target Length, A-P, and LR-axis on the mean amplitudes for words in the time window from 200 to 300 ms showed a significant interaction between Relatedness and A-P [*F*_(1, 14)_ = 15.76, *p* = 0.001, η^2^_*p*_ = 0.529] as well as a three-way interaction between Relatedness, A-P and LR-axis [*F*_(2, 28)_ = 5.45, *p* = 0.009, η^2^_*p*_ = 0.280, *GG* = 0.996; see Figure 2]. Related conditions showed more negative mean amplitudes at anterior sites than unrelated conditions, and this pattern was reversed at posterior sites. The three-way interaction revealed that the difference in μV between related and unrelated conditions was most pronounced at left-anterior electrodes (0.76). Comparing related and unrelated conditions, all differences were significant (Fischer's LSD = 0.206) except for the anterior-central (0.10) electrodes (see Figure 2). The localisation and the polarity of this effect fits best with a P350.

The analysis also showed a main effect of Syllabic Match [*F*_(1, 14)_ = 8.20, *p* = 0.013, η^2^_*p*_ = 0.369]. Collapsed over related and unrelated primes, “matching” conditions yielded more positive mean amplitudes (mean: 0.048 μV, SD: 0.929) than “mismatching” conditions (mean: −0.015 μV, SD: 0.973). Given that Syllabic Match only applies to related primes, and despite the fact that the interaction between Syllabic Match and Relatedness was not significant [*F*_(1, 14)_ = 1.90, *p* = 0.189], we computed separate ANOVAs on related and unrelated prime-target pairs. The ANOVA on related prime-target pairs showed an effect of Syllabic Match [*F*_(1, 14)_ = 6.88, *p* = 0.020, η^2^_*p*_ = 0.330]. Matching prime target pairs (mean: 0.053, SD: 0.150) were more positive than mismatching ones (mean: −0.041, SD: 0.106). Although in the same direction, (0.042 μV for “matching” pairs, 0.011 μV for “mismatching” pairs), the effect failed significance in the ANOVA on unrelated prime-target pairs [*F*_(1, 14)_ = 1.23, *p* = 0.285].

**Figure 3:**
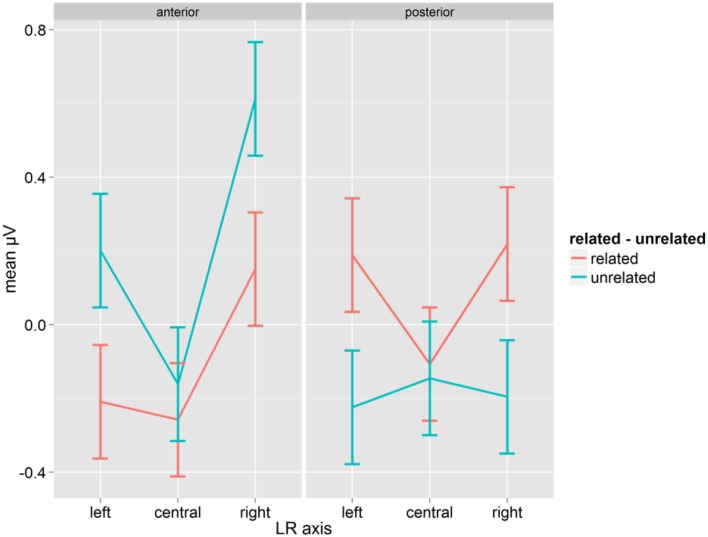
**Mean μV for pseudowords as a function of conditions for the time-window 200–300 ms; error bars represent Fisher's least significant differences**.

The analysis of pseudoword reactions showed no effects or interactions of Relatedness and Syllabic Match, neither overall nor in separate ANOVAs on related and unrelated prime-target pairs (all *p* > 0.12). However, the interaction of Relatedness, A-P and LR-axis was significant [*F*_(2, 28)_ = 15.11, *p* = 0.001, η^2^_*p*_ = 0.519, *GG* = 0.889, see Figure 3], with a very similar pattern to the P350 found for words. Fischer's LSD for the contrast of related and unrelated conditions was 0.153, and differences were significant except at anterior and posterior central sites (see Figure 3).

### Time window 300–400 ms

**Figure 4:**
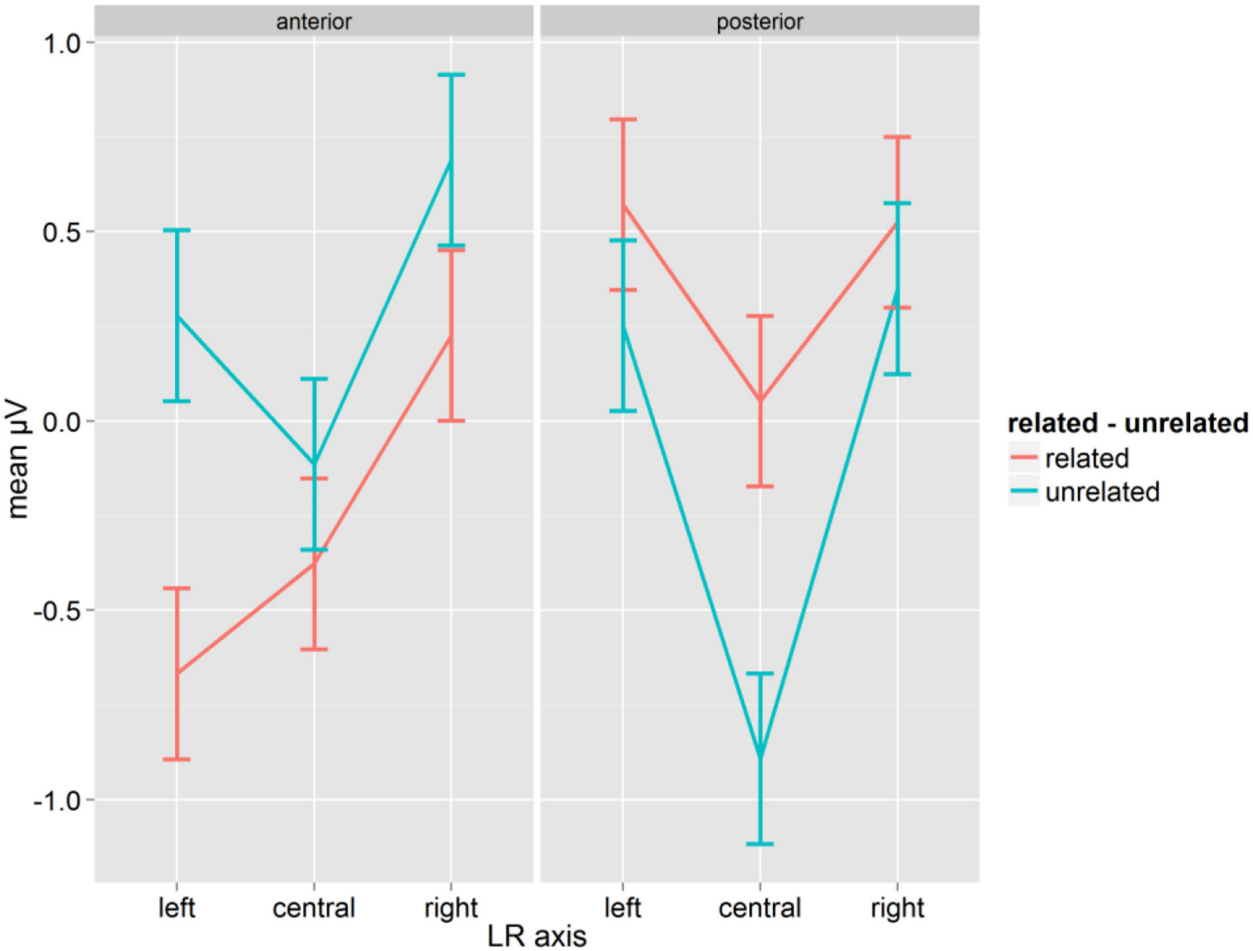
**Mean μV for words as a function of conditions for the time-window 300–400 ms; error bars represent Fisher's least significant differences**.

The same ANOVA as before was run for correct word responses using the mean amplitude in a time window of 300–400 ms. There was a significant main effect Relatedness [*F*_(1, 14)_ = 5.22, *p* = 0.038, η^2^_*p*_ = 0.271]. Overall, the amplitude of the related condition (mean: 0.055, SD: 1.14) was less positive than the amplitude of the unrelated condition (mean: 0.094, SD: 1.10). Relatedness interacted with A-P and with LR-axis, and the three-way interaction between Relatedness, A-P and LR-axis was also significant [*F*_(2, 28)_ = 4.96, *p* = 0.014, η^2^_*p*_ = 0.261, *GG* = 0.912; see Figure 4]. The mean amplitudes in the related condition were more negative than in the unrelated condition at anterior sites, and this pattern was reversed at posterior sites. The effect (related–unrelated) was most pronounced at left-anterior and central-posterior sites (0.94 for both), and much smaller (or insignificant, see Figure 4; Fischer's LSD = 0.225) at other sites. As with the earlier window, this pattern corresponds best with a P350. There was also a clear reversed effect (unrelated more negative than related) at posterior central electrodes, which seems indicative of an N400.

Collapsed over related and unrelated conditions, there was a main effect of Syllabic Match [*F*_(1, 14)_ = 4.95, *p* = 0.043, η^2^_*p*_ = 0.261; match mean: 0.095, SD: 1.14; mismatch mean: 0.053, SD: 1.10]. As for the 200–300 time window, we analyzed related and unrelated prime-target pairs separately, because Syllabic Match is a dummy variable for unrelated trials. The effect of Syllabic Match, with matching targets showing a more positive mean amplitude than mismatching targets (match mean: 0.09. mismatch mean: 0.01) just failed significance in the ANOVA on related prime-target pairs [*F*_(1, 14)_ = 4.42, *p* = 0.054, η^2^_*p*_ = 0.240]. Syllabic Match was not significant for unrelated prime-target pairs (*F* < 1).

The ANOVA on pseudowords yielded a significant interaction between Relatedness and A-P [*F*_(2, 28)_ = 17.09, *p* = 0.001, η^2^_*p*_ = 0.549], but the three-way interaction with LR-axis was not significant. As with words, mean amplitudes were more negative in related (−0.40) than in unrelated (0.01) conditions at anterior regions, while the reverse (related: 0.52; unrelated 0.16) was true at posterior regions (both effects are significant; Fischer's LSD = 0.277). As for the earlier time window, the polarity fits well with an anterior P350 effect. There were no effects of Syllabic Match for pseudoword targets in this time window, not in the overall analysis, nor in the analyses on related and unrelated trials separately (all *F* < 1).

### Time window 280–500 ms

Visual inspection showed a N400 at central-posterior electrodes, when comparing word targets preceded by related and unrelated prime fragments, with and without Syllabic Match (see Appendix 3: Figures 1, 2 in Supplementary Material). Based on the literature, we calculated the mean amplitude of a region of interest consisting of the electrodes C1, Cz, C2, CP1, CPz, CP2, P1, Pz, and P2 in a time window of 280–500 ms^2^. This served as the dependent measure in a Three-Way repeated measurement ANOVA with the factors Relatedness (related vs. unrelated), Lexicality (word vs. pseudoword), and Syllabic Match (present vs. absent in related conditions). No Greenhouse-Geisser-correction was needed because all factors had only two levels.

The analysis revealed a significant main effect of Relatedness [*F*_(1, 14)_ = 37.24, *p* = 0.001, η^2^_*p*_ = 0.727]. The mean amplitude in unrelated conditions was more negative (mean: −1.034, SD: 0.686) than in related conditions (mean: −0.464, SD: 0.745). Importantly, the interaction of Relatedness and Lexicality was also significant [*F*_(1, 14)_ = 12. 51, *p* = 0.003, η^2^_*p*_ = 0.472]. With Fischer's LSD of 0.23, related words (mean: −0.293, SD: 0.746) and related pseudowords (mean: −0.635, SD: 0.803) were reliably less negative than their unrelated counterparts (words mean: −1.133, SD: 0.900; pseudowords mean: −0.936, SD: 0.538). However, this difference —the N400 effect—was almost four times as large for words as for pseudowords. Furthermore, the interaction of Syllabic Match and Lexicality [*F*_(1, 14)_ = 5.37, *p* = 0.036, η^2^_*p*_ = 0.277] was significant. Collapsed over Relatedness, amplitudes to words were more negative with “matching” than with “mismatching” syllables, and this was reversed for pseudowords. Given that these data include the control trials to which syllabic match does not apply, separate analyses were calculated for related and unrelated prime-target pairs, for words and pseudowords separately. There were no effects of syllabic match, neither for related nor for unrelated prime-target pairs.

To summarize the ERP data: except for the earliest time widows (100–200 ms), there is clear evidence for effects of the relatedness between fragment primes and targets. We also observed main effects of syllabic match. Given that control primes neither share segments with, nor match the (abstract) syllabic structure of, the targets, main effects of syllabic match most probably reflect a correspondence in length of fragments and targets (long/long and short/short < long/short and short/long)—independent of phonological relatedness. However, such effects do not elucidate the impact of segmental and/or syllabic overlap between related primes and targets. Of interest for this question are main effects of relatedness between primes and targets, and interactions between relatedness and the other factors—most importantly syllabic match. Despite the fact that we found no significant interactions in our EEG data, we performed separate analyses on related and unrelated prime-target pairs. In the 200–300 ms window, there was an effect in related conditions. Targets whose first syllable corresponded to the prime fragment (e.g., /lus/ – /lus.tig/) elicited more positive values than targets preceded by fragments that mismatched their first syllable (e.g., /lus/ – /lust.los/). There was a similar trend in the 300–400 ms window. Unrelated pairs and pseudoword conditions showed no such effects in any time window.

With respect to the overall impact of related primes, a very similar pattern was observed in the two time windows lasting from 200–300 to 300–400. Related conditions showed more negative amplitudes than unrelated ones at anterior sites, but more positive amplitudes than unrelated ones at posterior sites. The strongest effects were observed over left-anterior electrodes. Clearly, this is not an N400 type effect. Given its left-anterior dominance, time window (200–400 ms), and polarity, the observed effect fits best with a modulation of the P350. Interestingly, these P350 effects were not qualified by an interaction with syllabic match. Thus, these differences between related and unrelated conditions held for all cases of overlap—syllabic or not. Note also that very similar patterns were observed for word and pseudoword targets in these time windows. The 300–400 ms window also revealed a pronounced central negativity for word trials, with more negative amplitudes for unrelated than for related conditions. This is similar to the central negativity observed by others (Friedrich et al., 2009; Schild et al., 2012). Given that the 300–400 time window largely overlaps with the time window from 280 to 500, we believe that this central negativity in fact corresponds to an N400.

The analysis on the 280–500 ms time window, on a selection of electrodes often used for N400-analyses, showed the expected difference between related and unrelated targets in the N400 time window. The N400 was more negative in unrelated than in related fragment conditions. This was qualified by an interaction, showing that this N400 effect was much larger for words than for pseudowords. No effects of syllabic match were observed in the N400 time window.

None of the EEG data revealed effects of syllabic match in interaction with relatedness. If related fragment primes that match the first syllable of the target had a special status, this should have revealed itself in such an interaction, because the control primes did not match the (abstract) syllabic structure. The only reliable evidence for an impact of syllabic match in related fragment-target pairs was observed in the 200–300 window; the 300–400 ms window showed a trend. Interesting as they are, these effects should be treated with caution since they did not reveal themselves in an interaction between syllabic match and relatedness, but in separate analyses of related und unrelated fragment conditions. Thus, whereas related prime fragments consistently have a different impact on ERP components than unrelated fragments from 200 ms onwards, this impact is not qualified by syllabic match, aside from the indication for related prime-target pairs in one time window.

## General discussion

This study used fragment priming, with related (e.g., /mu/) and unrelated (e.g., /tes/) spoken fragments to spoken targets words (e.g., /mu.tig/) or pseudowords. Relatedness was further varied along the dimension of syllabic match between fragments and targets. Whereas /mu/ specifies the initial segments of both /mutig/ and /mutlos/, it corresponds to the first syllable of /mutig/, not of /mutlos/. Syllabic match was implemented in related word and pseudoword conditions. Whereas obviously, all word targets were combined with existing syllables, the pseudoword targets that were phonotactically legal but not very word-like, were paired with fragments that, according to the rules of syllabification of German, structurally corresponded to their first syllables, but that in most cases were not part of the syllable inventory of the language.

First, we expected effects of relatedness, with an advantage (in RT) and differences (in EEG amplitude) between related and unrelated conditions. Second, if syllables play a role in German speech perception, related primes that precisely match the initial syllable should be superior to primes that match an equivalent number of initial phonemes but do not correspond to the first syllable. Third, differences in effects for words and pseudowords may inform us about the origin of effects of overlap. If words and pseudowords show similar effects, these may well originate from prelexical or early lexical levels of processing. If effects diverge, this indicates lexical involvement. Finally, a comparison of behavioral and ERP data, and of early and late EEG effects, may be informative with respect to the automaticity of potential effects, and on their dependence on advanced lexical processing. What do the data tell us, and how do they compare to results from other studies, in particular from the—admittedly small number of—studies that use the same paradigm and measures? We discuss the behavioral and EEG data separately.

### Reaction times in auditory lexical decision

To start with the behavioral data: There are effects of overlap for both words and pseudowords. For pseudowords, segmental overlap slows down lexical decision, for words, overlap speeds up reactions. Moreover, an effect of syllabic match of related fragments is present for existing words, most strongly in one particular condition. For pseudowords, overlap between fragments and targets slows down correct decisions, This may come as a surprise, since many fragments of pseudowords were not very wordlike by themselves. Note that their onsets (the first two or three segments), are compatible with existing words in the language and would thus activate lexical cohorts (Zwitserlood, 1989). In cases of segmental overlap between fragments and targets, this lexical activation may have interfered with a correct pseudoword decision on the targets. Moreover, the interference effect seems dependent on syllabic match between related primes and targets (in the analysis of the first-block data). This dependence on syllabic match of both the facilitation, with words, and the interference, with pseudowords, indicates a sensitivity to the syllabic structure of the targets that results in speeded word decisions and slowed pseudoword decisions. A likely locus for such a pattern is a postlexical, strategic one. Auditory lexical decision thus seems to tap into late effects of the syllabic match between prime fragments and their related targets, and may not be ideally suited to investigate the role of syllables during early phases of speech processing. The same has been argued for the monitoring task when it uses catch trials to prevent very fast decisions (see Zwitserlood, 2003; Floccia et al., 2012). In all, positive effects of syllabic match were not overly strong in the lexical decision task. Moreover, it is surprising that fragments that constituted the onsets of target words, but did not correspond to the first syllable, induced no facilitation at all (cf. Zwitserlood, 1989, 1996).

How do these behavioral data compare to those obtained by Friedrich and colleagues, in similar auditory-auditory fragment priming studies with lexical decision (Friedrich et al., 2009; Schild et al., 2012)? First, no data on pseudoword trials were reported in any of these studies. For word targets, both studies obtained significant facilitation, comparing latencies to targets after related and unrelated fragment primes. Taking a closer look at their materials, these fragments always corresponded to the first syllable of the spoken words. Thus, our behavioral data replicate effects reported for words in these studies, using the same paradigm to study the same language, German. Since both studies also registered EEG, it will be interesting to compare the ERP effects reported next.

### ERP data

The ERP data show no effects in the earliest time window (100–200 ms) that includes the N100. This is different from both studies that used auditory-auditory priming (Friedrich et al., 2009; Schild et al., 2012), who reported N100 or T-complex/N100 effects as a function of relatedness between primes and targets in this window. We find no such effects, neither for words nor for pseudowords. Given that these early effects are generally not the most robust, this might be due to the smaller number of participants remaining in the EEG analyses in our study (15 vs. 22 in the other studies).

In the two consecutive windows (200–300 and 300–400 ms), we observe clear modulations of the P350. The polarity of effects as well as the affected electrodes fits with what is observed by others: in (left) anterior regions, related trials show a more negative P350 than unrelated trials (cf. Friedrich et al., 2004a, 2008; Pylkkänen and Marantz, 2003, for the equivalent component from MEG). The P350 is taken to be sensitive to the degree of prime-target overlap. This fits well with the effects observed here, for both words and pseudowords. The P350 is also interpreted to reflect the activation of word-form representations. A related fragment facilitates lexical access relative to an unrelated fragment (see Friedrich et al., 2013). Note again that we obtain quite similar results for word and pseudowords targets. Given that pseudowords have no lexical representation, how can the P350 reflect facilitated lexical access? It should be noted that the first two, and often the first three, segments of the pseudoword primes and targets still correspond to existing words. Given the timing of the P350 and the moment in time at which spoken targets become pseudowords, it is quite feasible that their pseudoword status is not yet available to influence the phonological matching and word-form activation effects that are present and reliable throughout (see Friedrich et al., 2004a, for similar P350 effects with visual pseudoword targets).

In the N400 window, already present as a central negativity in the 300–400 ms window, the polarity of the relatedness effect reverses, with unrelated conditions being more negative than related conditions (see Appendix 3: Figures 1, 2 in Supplementary Material). This is in accordance with data for segmental overlap from many other studies (e.g., Praamstra et al., 1994; Dumay et al., 2001; Diaz and Swaab, 2007; Desroches et al., 2009; Scharinger and Felder, 2011). The N400 revealed an interaction between lexical status and relatedness. The N400, in terms of the difference between related and unrelated prime conditions, was much larger for words than for pseudowords—although the N400 for pseudowords was also reliable. In the N400 time window, lexical influences thus start to kick in, modulating the impact of form overlap between fragment primes and targets. Given that the time window extends to 500 ms, the information as to whether target stimuli are words or pseudowords should have become available for most stimuli. The pattern found for the N400 suggests that lexical selection is well on its way.

Given that we set out to investigate effects of syllabic match between fragment primes and targets, it is revealing that none of the EEG data revealed effects of syllabic match in interaction with relatedness. Only when—despite the lack of such interactions—related and unrelated conditions were analyzed separately did we observe an effect of syllabic match between prime fragments and word targets. As this is not statistically backed up by appropriate interactions, we feel somewhat reluctant to interpret this observation. Evidently, more research is needed to elucidate these effects. In sum, whereas related prime fragments have a different impact on the ERP components than unrelated fragments in all but the earliest time windows, effects of syllabic match are ephemeral—and definitely absent in the N400 window. It is also noteworthy that the time windows prior to the N400 showed effects to be very similar for words and pseudowords, most probably because their lexical status is not yet clear. What the ERP data reveal, are the processes involved in phonetic/phonological matching, lexical access and selection during spoken-word recognition. The consistent advantage of related fragments for target processing, including the mapping of incoming speech, lexical access and selection, fits many models of spoken word recognition (McClelland and Elman, 1986; Marslen-Wilson, 1987; see also Zwitserlood, 1989). The obvious conclusion is that syllabic match is not crucial for these processes. Our data provide no support for syllable-sized prelexical representations that mediate between the speech input and the mental lexicon. Note that syllabic cues may still play an important role in speech segmentation; our fragment-priming paradigm does not really address this question (cf. Zwitserlood, 2003). Why, then, do lexical decision latencies show at least some effects of syllabic match? The most tempting interpretation is that syllabic effects obtained in behavioral data, such as the lexical decision latencies reported here, reflect either late lexical processing or even post-lexical strategic processing, but not speech perception and lexical access. This is supported by the fact that we found no evidence for syllabic match in reaction times to pseudowords. Note that the behavioral data are somewhat puzzling to start with, with no evidence of morphological priming between the longer fragments and both related targets. This pattern indicates a dissociation of more automatic processes—evident in the ERPs—and data from tasks that require conscious target processing—such as lexical decision. Such dissociations are an all-to-familiar phenomenon in research on early processes in speech perception (cf. Bien and Zwitserlood, 2013). With respect to the quest for the syllable as a prelexical unit of speech processing, the following quote elegantly sums up the problem: “In sum, although a lot of evidence indicates that the syllabic structure influences spoken word recognition, there is very little support for the idea that syllabic coding units are extracted from the signal and intervene in the perceptual processes” (Dumay and Content, 2012, p. 682).

### Conflict of interest statement

The authors declare that the research was conducted in the absence of any commercial or financial relationships that could be construed as a potential conflict of interest.

## Footnotes

^1^The dot marks a syllable boundary.

^2^Using a time-window of 350 ms to 450 ms yielded the same significant main effect of Relatedness [*F*_(1, 14)_ = 40.949, *p* = 0.001, η^2^_*p*_ = 0.745] and the significant interaction of Relatedness and Lexicality [*F*_(1, 14)_ = 12.599, *p* = 0.003, η^2^_*p*_ = 0.474). The interaction of Syllabic Match and Lexicality [*F*_(1, 14)_ = 2.745, *p* = 0.120, η^2^_*p*_ = 0.164], however, was not significant.
