# Supplementary material for: Do syllables play a role in German speech perception? Behavioral and electrophysiological data from primed lexical decision
Source: Front Psychol. 2015 Jan 12;5:1544. doi: 10.3389/fpsyg.2014.01544 (PMC4290540; doi:10.3389/fpsyg.2014.01544)
Supplement: Supplementary file 1 [file DataSheet1.DOCX]

Appendix 1

Table 1: Targets mean duration in milliseconds, mean pitch in Hz and mean intensity in db (SD in parentheses)

|  |  | target length | | | | | |
| --- | --- | --- | --- | --- | --- | --- | --- |
|  |  | long | | | short | | |
| lexical status | | duration | pitch | intensity | duration | pitch | intensity |
|  | word | 917 (85) | 236 (10) | 57 (3) | 817 (76) | 244 (12) | 58 (4) |
|  | pseudoword | 999 (63) | 240 (12) | 56 (3) | 895 (66) | 239 (12) | 55 (3) |

Note: Targets were repeated over the factors syllabic match and relatedness. Therefore, figures were not broken down with respect to these factors.

Table 2: Primes mean duration in milliseconds, mean pitch in Hz and mean intensity in db (SD in parentheses)

|  |  | length | | | | | |
| --- | --- | --- | --- | --- | --- | --- | --- |
|  | |  | long |  |  | short |  |
| prime type | | duration | pitch | intensity | duration | pitch | intensity |
|  |  |  |  | word target | |  |  |
|  | related | 612 (76) | 220 (14) | 57 (4) | 535 (77) | 225 (15) | 60 (4) |
|  | unrelated | 629 (80) | 223 (19) | 57 (4) | 564 (86) | 220 (19) | 59 (3) |
|  | |  |  | pseudoword target | |  |  |
|  | related | 636 (65) | 229 (15) | 57 (4) | 547 (58) | 233 (19) | 61 (3) |
|  | unrelated | 667 (72) | 226 (16) | 56 (4) | 559 (51) | 230 (17) | 30 (4) |

Note: Primes were repeated over the factor syllabic match. Therefore, figures are not broken down with respect to this factor.

Appendix 2

Table 1: Percentage of artifacts and SD (in parentheses) as a function of condition

|  |  |  | Target Length | |
| --- | --- | --- | --- | --- |
| Lexicality | Relatedness | Syllabic Match | Short | Long |
| Word |  |  |  |  |
|  | Related | + | 10 (8.7) | 12 (10.6) |
|  |  | - | 12 (9.9) | 9 (7.1) |
|  | Unrelated | (+) | 10 (7.1) | 12 (8.0) |
|  |  | (-) | 13 (9.2) | 10 (9.8) |
| Pseudoword |  |  |  |  |
|  | Related | + | 12 (7.4) | 11 (9.6) |
|  |  | - | 8 (8.2) | 10 (8.3) |
|  | Unrelated | (+) | 13 (8.2) | 11 (6.8) |
|  |  | (-) | 8 (7.8) | 10 (9.4) |

Appendix 3


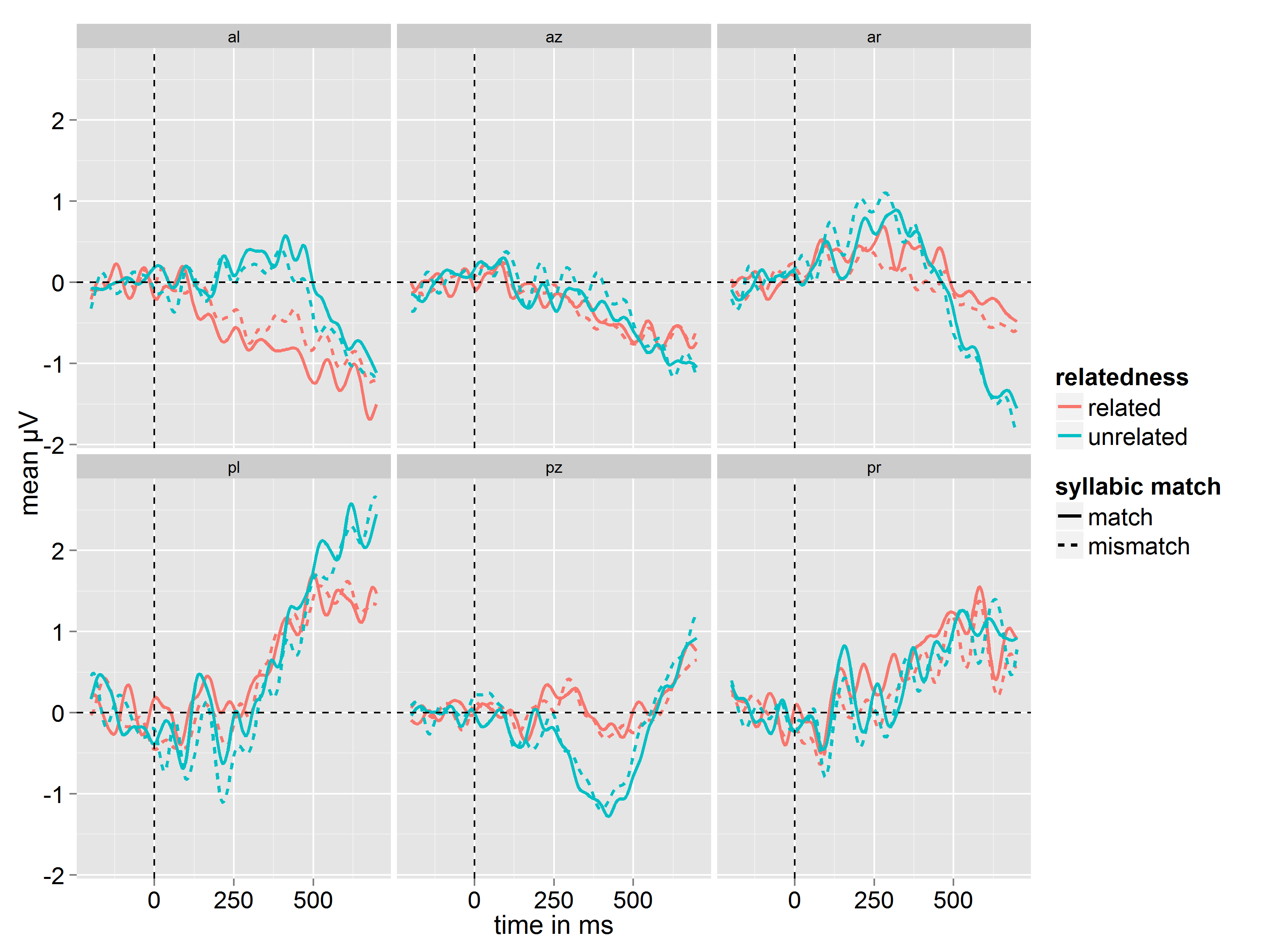


Figure 1: Mean voltage as function of RELATEDNESS, SYLLABIC MATCH and REGION OF INTEREST for words.

Note: al = anterior-left, az: anterior-central, ar: anterior-right, pl: posterior-left, pz: posterior-central, pr: posterior-right


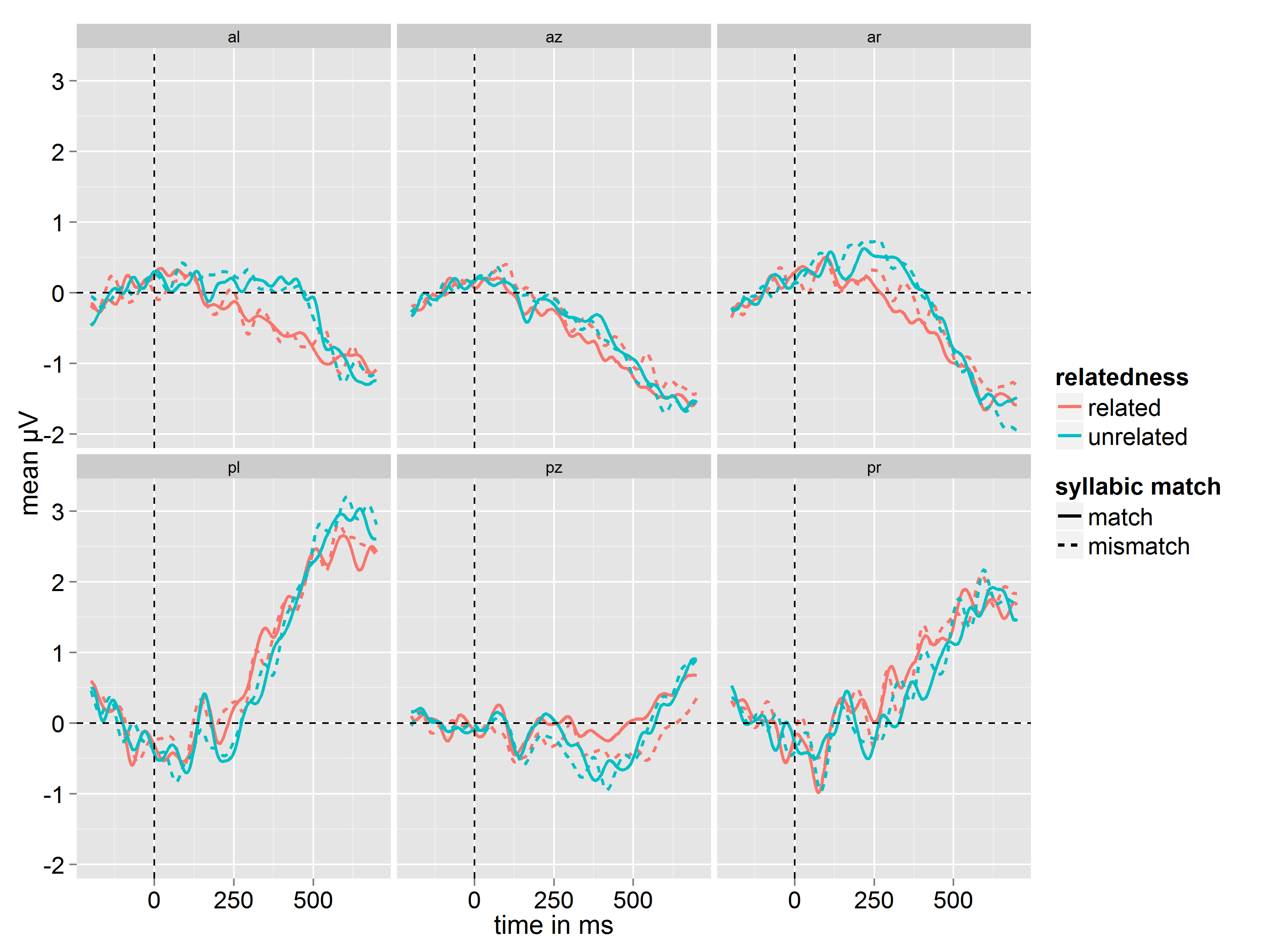


Figure 2: Mean voltage as function of Relatedness and Region of Interest for pseudowords.

Note: al = anterior-left, az: anterior-central, ar: anterior-right, pl: posterior-left, pz: posterior-central, pr: posterior-right

**Appendix 4: Stimuli**

Table 1: Prime-target combinations long targets

|  | relatedness | | | |
| --- | --- | --- | --- | --- |
|  | related | | unrelated | |
|  | syllabic match | | syllabic match | |
| target | + | - | + | - |
| blutbild | blut | blu | flut | flu |
| bratfisch | brat | bra | duld | dul |
| dankbar | dank | dan | glaub | glau |
| deutlich | deut | deu | blas | bla |
| dichtkunst | dicht | dich | macht | mach |
| greifarm | greif | grei | west | wes |
| haftbar | haft | haf | flaum | flau |
| heilbar | heil | hei | kind | kin |
| hörspiel | hör | hö | rind | rin |
| kreuzfahrt | kreuz | kreu | sond | son |
| laufband | lauf | lau | wald | wal |
| lösbar | lös | lö | brust | bru |
| luftleer | luft | luf | zaun | zau |
| lustlos | lust | lus | trag | tra |
| mutlos | mut | mu | test | tes |
| nervlich | nerv | ner | mark | mar |
| planlos | plan | pla | streich | strei |
| prüfbar | prüf | prü | gras | gra |
| rauschgift | rausch | rau | hals | hal |
| reizbar | reiz | rei | hüft | hüf |
| saftlos | saft | saf | grün | grü |
| salzlos | salz | sal | kran | kra |
| scheinbar | schein | schei | spät | spä |
| schreibtisch | schreib | schrei | rand | ran |
| sichtbar | sicht | sich | haus | hau |
| spürbar | spür | spü | kron | kro |
| suchbar | such | su | fran | fra |
| taktvoll | takt | tak | rund | run |
| tankwart | tank | tan | recht | rech |
| tastbar | tast | tas | klug | klu |
| tauschwert | tausch | tau | rost | ros |
| teilbar | teil | tei | licht | lich |
| tröstlich | tröst | trö | hand | han |
| witzlos | witz | wit | schraub | schrau |
| zahlbar | zahl | zah | nest | nes |

Table 2: Prime-target combinations short word targets

|  | relatedness | | | |
| --- | --- | --- | --- | --- |
|  | related | | unrelated | |
|  | syllabic match | | syllabic match | |
| target | + | - | + | - |
| bluten | blu | blut | flu | flut |
| braten | bra | brat | dul | duld |
| dankend | dan | dank | glau | glaub |
| deutung | deu | deut | bla | blas |
| dichten | dich | dicht | mach | macht |
| greifen | grei | greif | wes | west |
| haftung | haf | haft | flau | flaum |
| heilung | hei | heil | kin | kind |
| hören | hö | hör | rin | rind |
| kreuzen | kreu | kreuz | son | sond |
| laufen | lau | lauf | wal | wald |
| lösen | lö | lös | bru | brust |
| luftig | luf | luft | zau | zaun |
| lustig | lus | lust | tra | trag |
| mutig | mu | mut | tes | test |
| nervig | ner | nerv | mar | mark |
| planung | pla | plan | strei | streich |
| prüfung | prü | prüf | gra | gras |
| rauschen | rau | rausch | hal | hals |
| reizen | rei | reiz | hüf | hüft |
| saftig | saf | saft | grü | grün |
| salzig | sal | salz | kra | kran |
| scheinend | schei | schein | spä | spät |
| schreiben | schrei | schreib | ran | rand |
| sichtend | sich | sicht | hau | haus |
| spürend | spü | spür | kro | kron |
| suchen | su | such | fra | fran |
| takten | tak | takt | run | rund |
| tanken | tan | tank | rech | recht |
| tastend | tas | tast | klu | klug |
| tauschen | tau | tausch | ros | rost |
| teilen | tei | teil | lich | licht |
| trösten | trö | tröst | han | hand |
| witzig | wit | witz | schrau | schraub |
| zahlend | zah | zahl | nes | nest |

Table 3: Prime-target combinations long pseudoword targets

|  | relatedness | | | |
| --- | --- | --- | --- | --- |
|  | related | | unrelated | |
|  | syllabic match | | syllabic match | |
| target | + | - | + | - |
| bentlok | bent | ben | womp | wom |
| dostwik | dost | dos | milp | mil |
| fostlak | fost | fos | samp | sam |
| gantlok | gant | gan | fimp | fim |
| gemtmek | gemt | gem | gulp | gul |
| gintlech | gint | gin | drup | dru |
| gontmak | gont | gon | jelp | jel |
| gostmek | gost | gos | melp | mel |
| hamplech | hamp | ham | sukt | suk |
| jolplos | jolp | jol | nant | nan |
| jultmok | jult | jul | donk | don |
| kimplech | kimp | kim | quast | quas |
| kostbar | kost | kos | stun | stu |
| lentmok | lent | len | bolp | bol |
| limpmoll | limp | lim | makt | mak |
| lompmak | lomp | lom | kunt | kun |
| luntmek | lunt | lun | sosk | sos |
| mektpall | mekt | mek | himp | him |
| mimplech | mimp | mim | mest | mes |
| muktvill | mukt | muk | bemp | bem |
| nemplech | nemp | nem | muls | mul |
| neunfach | neun | neu | wein | wei |
| pontmak | pont | pon | sulm | sul |
| pulmvoll | pulm | pul | drap | dra |
| puntmek | punt | pun | gomp | gom |
| ristlas | rist | ris | somp | som |
| rontlok | ront | ron | selp | sel |
| rustwak | rust | rus | semp | sem |
| schentmak | schent | schen | rimp | rim |
| senzlach | senz | sen | kulp | kul |
| siltlok | silt | sil | malp | mal |
| suntmik | sunt | sun | wolm | wol |
| tentlech | tent | ten | relp | rel |
| tiltlich | tilt | til | moks | mok |
| tulftig | tulf | tul | tekt | tek |
| ventlak | vent | ven | durp | dur |
| wostlech | wost | wos | jilp | jil |
| zastmok | zast | zas | kelk | kel |
| zustvoll | zust | zus | memp | mem |

Table 4: Prime-target combinations short pseudoword targets

|  | relatedness | | | |
| --- | --- | --- | --- | --- |
|  | related | | unrelated | |
|  | syllabic match | | syllabic match | |
| target | + | - | + | - |
| bento | ben | bent | wom | womp |
| dostig | dos | dost | mil | milp |
| fostig | fos | fost | sam | samp |
| gantech | gan | gant | fim | fimp |
| gemten | gem | gemt | gul | gulp |
| gintig | gin | gint | dru | drup |
| gontig | gon | gont | jel | jelp |
| gostech | gos | gost | mel | melp |
| hampig | ham | hamp | suk | sukt |
| jolpek | jol | jolp | nan | nant |
| jultep | jul | jult | don | donk |
| kimpod | kim | kimp | quas | quast |
| kostend | kos | kost | stu | stun |
| lentig | len | lent | bol | bolp |
| limpal | lim | limp | mak | makt |
| lompek | lom | lomp | kun | kunt |
| luntig | lun | lunt | sos | sosk |
| mektull | mek | mekt | him | himp |
| mimpig | mim | mimp | mes | mest |
| muktell | muk | mukt | bem | bemp |
| nempig | nem | nemp | mul | muls |
| neune | neu | neun | wei | wein |
| pontig | pon | pont | sul | sulm |
| pulmek | pul | pulm | dra | drap |
| puntech | pun | punt | gom | gomp |
| ristaft | ris | rist | som | somp |
| rontak | ron | ront | sel | selp |
| rustep | rus | rust | sem | semp |
| schentak | schen | schent | rim | rimp |
| senzill | sen | senz | kul | kulp |
| siltig | sil | silt | mal | malp |
| suntill | sun | sunt | wol | wolm |
| tentak | ten | tent | rel | relp |
| tiltich | til | tilt | mok | moks |
| tulfek | tul | tulf | tek | tekt |
| ventak | ven | vent | dur | durp |
| wostech | wos | wost | jil | jilp |
| zastig | zas | zast | kel | kelk |
| zusten | zus | zust | mem | memp |
